# Community health volunteers improve access to malaria case management in Siaya County, Kenya

**DOI:** 10.1186/s12936-026-05883-3

**Published:** 2026-03-31

**Authors:** Wycliffe Odongo, Victoria Seffren, Kizito Obiet, Brian Seda, Oliver Towett, Hong Zhou, Daniel P. McDermott, Simon Kariuki, Sarah G. Staedke, Feiko ter Kuile, Titus K. Kwambai, Aaron M. Samuels, Julie R. Gutman

**Affiliations:** 1 Malaria Branch, National Center for Emerging and Zoonotic Infectious Diseases, Centers for Disease Control and Prevention, Atlanta, GA USA; 2 Centre for Global Health Research, Kenya Medical Research Institute, Kisumu, Kenya; 3 Department of Vector Biology, Liverpool School of Tropical Medicine, Liverpool, UK; 4 Department of Clinical Sciences, Liverpool School of Tropical Medicine, Liverpool, UK; 5 Malaria Branch, National Center for Emerging and Zoonotic Infectious Diseases, US Centers for Disease Control and Prevention, Kisumu, Kenya

**Keywords:** Community health volunteers (CHVs), Community health workers, Malaria case management, Health system access, Community surveillance

## Abstract

Malaria remains a major health challenge in western Kenya, where transmission persists despite decades of control efforts. Community health volunteers (CHVs) are increasingly engaged in community case management (CCM) to expand timely malaria care, but quantitative evidence of their contributions is limited. This analysis evaluated the effectiveness of CHVs in malaria case management and their impact on healthcare access in Siaya County. Data from 1.4 million encounters recorded by 936 CHVs and 86 health facilities (HFs) across 713 villages (2021–2023) were analyzed. The outcomes included coverage, testing practices, test positivity rates (TPRs), malaria incidence, and adherence to treatment guidelines. CHV coverage was defined as adequate at one CHV per ≤ 500 people; HF access was estimated via the two-step floating catchment area (2SFCA) method, incorporating proximity and utilization, with access scores classified as adequate or inadequate. CHV coverage was adequate for 82.5% of the villages vs. 30% for HFs. CHVs managed 28.1% of all encounters, including 12.1% of suspected malaria cases and 15.6% of rapid diagnostic test–positive cases. A greater proportion of CHV-suspected patients reported fever (77.0% vs. 53.1%). HFs more suspected and febrile cases, but afebrile testing was similar. CHVs recorded higher TPRs than HFs did (82.0% vs. 64.3%). Nearly all confirmed malaria cases received antimalarials. Incorporating CHV data increased malaria incidence estimates by 18% (467 vs. 549 per 1,000 population). CHVs extend access to malaria diagnosis and treatment, particularly in areas with limited HF coverage. Their practices align with national guidelines, and program expansion with adequate support could enhance malaria control in resource-limited settings.

**Supplementary Information:**

The online version contains supplementary material available at 10.1186/s12936-026-05883-3.

## Introduction

Malaria remains a public health challenge in sub-Saharan Africa. In the Lake Endemic region of western Kenya, transmission persists despite the widespread scaleup of key malaria control interventions [1, 2]. Prompt care seeking for febrile illness - defined as seeking treatment within 24–48 h of symptom onset—is estimated at 62%, which is well below global targets, despite being critical for preventing severe disease and reducing transmission [3]. In this context, community health volunteers (CHVs) play an essential role in the fight against malaria as implementers of the community case management of malaria (CCM) strategy [4–9]. In Kenya, malaria testing occurs primarily at health facilities (HFs), supplemented by CHVs and private pharmacies [6, 10]. CHVs not only provide CCM services but also collect data on community health services.

CHVs expand access to malaria case management, especially in areas with limited access to HFs [11], and are integral to the World Health Organization (WHO)-endorsed CCM strategy aimed at reducing malaria morbidity and mortality, especially in children under five years of age [12]. Under this strategy, CHVs are equipped with rapid diagnostic tests (RDTs) and artemisinin-based combination therapies (ACTs) to manage uncomplicated malaria, and refer severe cases to higher-level HFs [11]. Studies from malaria-endemic countries have shown that the diagnostic accuracy of tests performed by CHVs is comparable to that of experienced laboratory personnel and other healthcare workers [6, 11, 13–15]. Additionally, CHVs collect and report data to the Ministry of Health (MoH) on community demographics, health status, and health services rendered.

Despite their critical role in malaria control, CHVs in Kenya work for ~ $35/month without a guaranteed minimum pay [16, 17]. While qualitative evidence highlights their contributions, existing research has largely neglected quantitative evaluations of their specific roles in case detection and management [18–20]. This knowledge gap limits evidence-based decision-making for scale-up and resource allocation [20, 21]. Integrating granular CHV data with HF data is crucial for monitoring malaria transmission dynamics and optimizing intervention strategies [22–24]. This study evaluated CHV contributions to malaria case detection and management in two subcounties of Siaya County, Kenya, providing quantitative evidence to inform policy and program implementation.

## Methods

### Study area and population

**Fig. 1:**
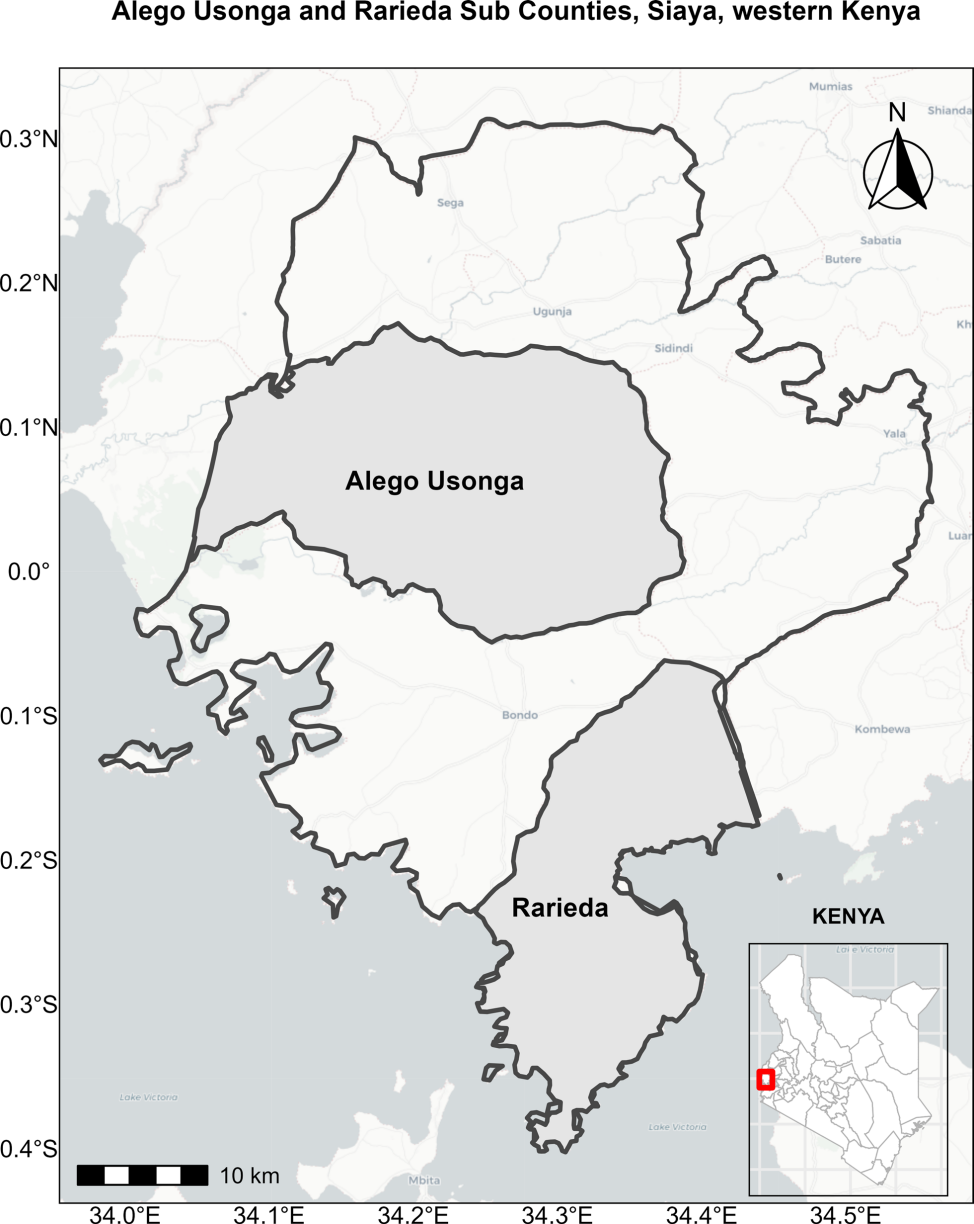
Geographic location of the study area in Alego Usonga and Rarieda subcounties within Siaya County, western Kenya. Map showing Siaya County (2,530 km^2^), with Alego Usonga (605 km^2^) and Rarieda (644km^2^, 38% covered by Lake Victoria) sub-counties. Both are among the six sub-counties in Siaya County

The study was conducted in Alego Usonga and Rarieda subcounties of Siaya County, western Kenya, from January 2021 to December 2023 (Fig. 1). Siaya County, spanning 2530 km^2^ with 993,183 residents [25], experiences year-round malaria transmission, with peaks during long (May–July) and short rains (October-December) [26, 27]. In 2020, 77.9% of households in the Lake Endemic Region owned at least one insecticide-treated bed net (ITN) [28]; in 2023, 88.5% of those in the study area [29].

#### Health systems structure:

Kenya's public health system has six hierarchical levels, with CHVs operating at Level 1 (community unit [CU]), linking communities to higher-level HFs (Levels 2–6). Each CHV serves ~ 500 people or 100 households [8, 17, 30]. CHVs are supervised by community health assistants (CHAs), each supervising ~ 25 CHVs affiliated with a HF [30, 31]. CHVs - now called community health promoters - conduct quarterly household visits for preventive services, referrals to HFs for illness and pregnancy, birth and death documentation, and carry out annual household registration of health and demographic data [8, 9, 32–34].

The study included 86 HFs and all the CUs (86) across two subcounties-47 CUs in Alego Usonga, 39 CUs in Rarieda-covering 713 villages with an average of 482 residents (5.3 per household), served by 936 CHVs. Each CU served 8–15 villages, averaging a catchment of 4,817 residents (IQR = [3,544, 5,566]) [2023 unpublished household registration data]. HFs comprised 62 Level 2 dispensaries, 22 Level 3 health centers, and 2 Level 4 subcounty hospitals, all of which utilized scannable forms (ScanForm) for data collection. These facilities are operated by public (76.7%), private (15.2%), and faith-based or nongovernmental organization (7.1%) sectors.

CCM is fully implemented across Siaya County, with CHVs delivering iCCM and referring cases to link HFs. In the national DHIS2 reporting system, CHV data are aggregated with link HF data, limiting visibility of CHV-specific contributions; cross-catchment care seeking and potential duplicate counting remain known constraints.

### Data collection

This study was conducted as part of a malaria surveillance strengthening initiative to improve routine reporting. Standard MoH outpatient department (OPD) and CHV registers were redesigned into scannable formats and digitized via ScanForm, a proprietary optical character recognition (OCR) system that converts handwritten records into digital data [35]. The malaria surveillance project was rolled out in phases, starting in January 2021 with 40 CUs reporting, expanding to 72 by June 2021, and 86 by October 2022. CHVs and HF staff continued routine CCM activities; no additional clinical services were introduced. Registers were scanned monthly by CHAs and project staff via the ScanForm mobile application. Monthly supervision supported scanning procedures, data quality review, and refresher training on indicators. Uploaded data underwent routine quality checks to ensure completeness prior to analysis. OPD registers were used to track patient encounters and malaria testing, whereas CHV registers documented malaria testing, household preventive and referral services and the demographics of household members (see supplemental materials for details captured on registers). Encounter-level data included age, sex, visit date, fever status, malaria test results, artemisinin-based combination therapy (ACT) treatment, and village of residence. To demarcate village boundaries, villages were mapped via geographical positioning system (GPS) devices during household registration in 2022. HF GPS coordinates and county shapefiles were obtained from Kenya MoH's Master Health Facility Registry (KMHFR) and Humanitarian Data Exchange [36]. Household registration provided village-level population data, whereas census data from the Kenya National Bureau of Statistics were used to validate subcounty and county administrative boundaries (villages, wards and sublocations) [37].

### Data analysis

#### Healthcare utilization:

HF data included a unique OPD encounter number, while CHV registers used a compound-household-individual ID; these were not harmonized. Shared village codes enabled village-level aggregation after appending datasets for encounter-level analysis. A composite key (source, visit date, village code, age, sex, test status, and result) flagged potential duplicates. Healthcare utilization was assessed by comparing the proportion of the population served by CHVs with that served by HFs. Patient-level health encounter data were aggregated by patient’s village of residence for CHVs and by village-facility pairs for HF data to reflect utilization levels. Village-level utilization was measured by the proportion of patient encounters each HF received from neighboring villages, defined as villages with at least 100 encounters per year. Malaria case management statistics, including test positivity rates (TPRs), incidence rates, and treatment rates, were calculated using epitools [38] and epiR packages in R (version 4.4.0). TPR was defined as the proportion of positive cases to total tests, incidence as new cases per total catchment population at risk, treatment rate as the proportion of positive cases receiving ACTs and suspected malaria as encounters with any of the following recorded: fever or a history of fever in the last 7 days, tested for malaria, or treated with ACTs.

#### Healthcare coverage:

Village boundary polygons and HF GPS coordinates were linked to aggregated CHV and HF data, respectively, by village IDs to enable Haversine distance [39] calculations and map coverage. CHV coverage analysis compared village-level CHV-to-population ratios against the MoH-recommended ratio of 1:500, categorizing villages with ratios of 1: ≤ 500 as “adequate” and those with ratios of 1: > 500 as “inadequate”.

HF coverage, defined as how easily village residents can reach a HF (distance) and how often they seek care there (utilization),was measured via the two-step floating catchment area (2SFCA) method [40], which incorporates proximity (distance and distance-decay effects), utilization, and catchment population. Village-to-nearest HF distances (range: 0.1–5.5 km) were calculated between village centroids (from boundary polygons) and HFs (GPS coordinates) via the Haversine formula (39) in R (geosphere package v1.5.20). A 10-km HF catchment threshold was applied to reflect travel distances in these semiurban and rural areas, consistent with prior studies. Catchment population was aggregated by village ID from household census data to represent local demand. A Gaussian distance-decay function then modeled the decreasing likelihood of HF attendance with increasing distance, assigning more weight to nearby villages [41]. Utilization proportions by villages within the catchment area were derived and included in the model to reflect actual care-seeking behavior [42]. HF accessibility scores were then calculated by integrating all three variables: catchment population, distance-decay and utilization. For each village, its population and proportion of care utilized at nearby HFs (demand) were used to weight each HF’s contribution to the accessibility score. These HF scores were summed by village, considering only HFs within a 10-km catchment radius, with each score further weighted by the village’s proximity to the HF and observed utilization, ensuring that villages near more frequently utilized and accessible HFs received proportionally higher accessibility scores. To avoid disproportionate influence from individual HFs, village accessibility scores were normalized. Higher scores indicate better access, whereas lower scores reflect limited proximity or utilization. The villages were then classified into “adequate” and “inadequate” HF coverage groups via the Jenks natural breaks method [43].

Finally, villages were classified into four groups based on the combined HF and CHV coverage status: both adequate, both inadequate, and adequate in one while inadequate in the other. The associations between incidence and coverage adequacy were evaluated via linear regression models stratified by CHV + HF and HF-only sources, with coverage adequacy as a categorical predictor. Mixed-effects models were employed to account for village-level clustering and coverage variation. Shapiro‒Wilk and Wilcoxon signed-rank tests were used to assess normality and distributional differences prior to model fitting.

#### Regression analysis:

A modified Poisson regression model was used to estimate adjusted risk ratios (aRRs) for factors associated with malaria RDT positivity. Standard errors were adjusted for clustering at the village level via the ‘*sandwich’* (v3.1) and ‘*lmtest’ (v0.9–40)* packages in R. Individual-level predictors included age, sex, fever history, year, provider type, and subcounty; village-level predictors included CHV and HF coverage. Distance and HF accessibility scores were excluded due to collinearity.

## Results

### Healthcare coverage

#### Community health volunteer coverage

**Fig. 2:**
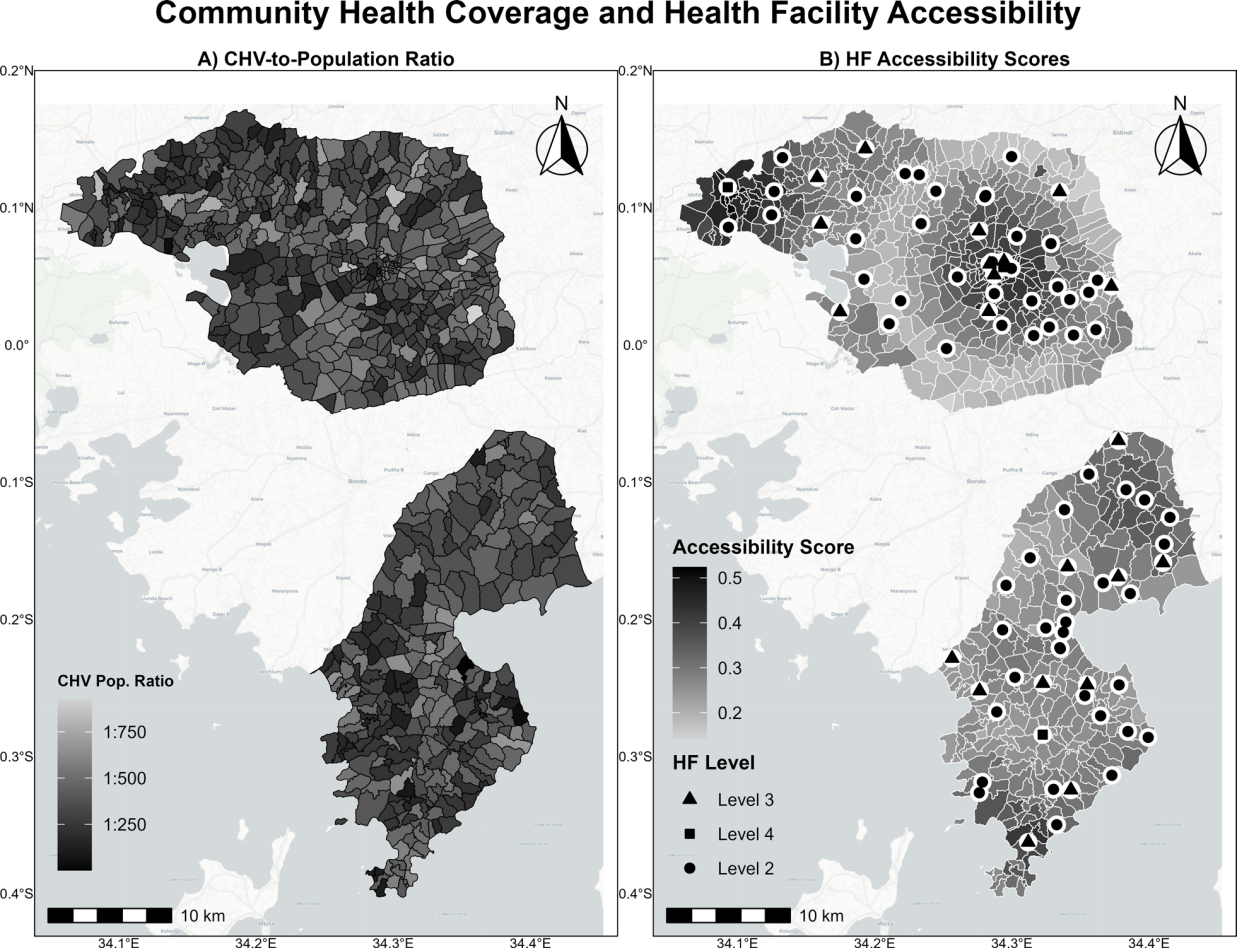
Community Health Volunteer (CHV) Coverage and Health Facility (HF) Accessibility in Alego Usonga and Rarieda Sub-Counties, Siaya, western Kenya (2021-2023). The maps highlight disparities in both CHV and HF coverage. Panel **A** shows the village-level CHV-to-population ratio, with darker shading indicating better CHV coverage per capita. Panel **B** displays spatial variation in HF accessibility scores, where higher scores represent better access to HF services. Health facility locations are marked by symbols denoting facility level (Level 2: dispensaries, Level 3: health centers, Level 4: hospitals). Health facility coverage was assessed using the Two-Step Floating Catchment Area method, which incorporates spatial proximity, facility attendance, and a Gaussian decay function to generate village-level accessibility scores

The MOH-recommended CHV coverage standard (1:500) was met in 82.5% (588/713) of the villages, with an average of 1:329 residents (Fig. 2). Conversely, 125 villages fell below the standard, with a mean CHV-to-population ratio of 1:585 (range: 502–926). Overall, the mean ratio was 1:375 (IQR = [273, 472]). Coverage was better in villages with multiple CHVs (1:318) than in those with a single CHV (1:412, p < 0.001).

During patient encounters captured in their registers (n = 417,340), CHVs provided multiple healthcare services, most commonly malnutrition monitoring (30.8%) and malaria care (27.4%), followed by family planning (FP) (13.2%), growth monitoring (12.9%), deworming (7.6%), and antenatal support for pregnant women (5.5%). Other combinations of maternal and child health services constituted 6.5% of encounters.

#### Health facility coverage

Although nearly all villages (711/713) met the WHO distance-based coverage standards (proximity to a HF ≤ 5 km), only 30.0% (n = 214) had adequate coverage based on modeled accessibility scores, representing 29.8% of the total population and revealing significant coverage gaps. The median Haversine distance to the nearest HF was 1.4 km (IQR [0.9, 2.0]; max: 5.5 km), with 2SFCA accessibility scores ranging from 0.143 (very inadequate) to 0.524 (very adequate) access (mean: 0.296; median: 0.282), indicating a fivefold disparity in accessibility (Fig. 2).

About 24.3% of the villages (n = 173) had adequate coverage by both CHVs and HFs, representing 22.4% of the total population (110,503 residents). In contrast, 11.8% (n = 84 villages) experienced inadequate coverage from both sources, accounting for 13.7% of the total population [50, 860 residents]. Mixed coverage was more common: 58.2% of villages (n = 415; 209,577 residents) had adequate CHV but inadequate HF coverage, whereas 5.8% (n = 41, 27,224 residents) had inadequate CHV coverage but adequate HF coverage (Table 1). Villages with adequate coverage by both were, on average, 1.0 km from the nearest HF, compared to 1.8 km in inadequately covered villages (p < 0.001).

**Table 1:** Village-level matrix of service coverage adequacy: comparing chvs and health facilities

HF coverage	CHV coverage		
	Adequate	Inadequate	Total
Adequate	173 (24.3%) 83,279 (22.4%)	41 (5.8%) 27,224 (7.3%)	214 (30.0%) 110,503 (29.8%)
Inadequate	415 (58.2%) 209,577 (56.5%)	84 (11.8%) 50,860 (13.7%)	499 (70.0%) 260,437 (70.2%)
Total	588 (82.5%) 292,856 (79.0%)	125 (17.5%) 78,084 (21.0%)	713 (100.0%) 370,940 (100.0%)

Each cell shows; Top indicates count of villages and the % of the total (713 villages); Bottom shows the population and % of the total population

CHV coverage was defined as a CHV-to-population ratio of 1: ≤ 500; HF coverage was estimated using the 2SFCA method

### Healthcare utilization

**Fig. 3:**
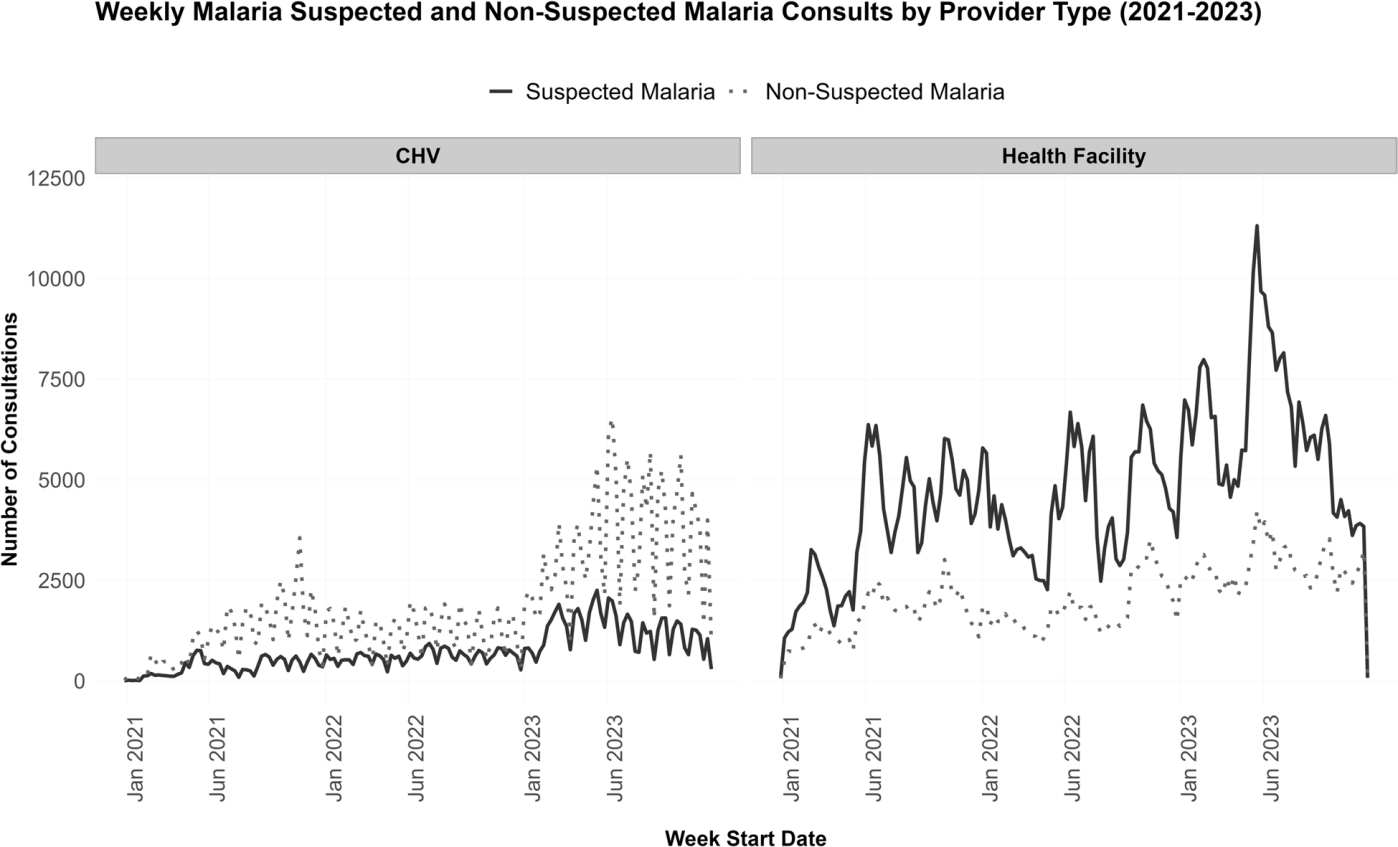
Weekly malaria suspected and non-suspected malaria consults by provider type (2021-2023). The plots show the number of suspected and non-suspected malaria consultations documented by community health volunteers (CHV) and health facilities from January 2021 to December 2023, in Alego and Rarieda Sub-Counties, Siaya, western Kenya. Suspected malaria cases (solid line) are defined as individual who reported history of fever or exhibited malaria-related symptoms (clinical malaria), or were tested for malaria, or received treatment. Non-suspected malaria consultations (dashed line) represent all other consultations. Data excludes the incomplete week ending December 31, 2023

Level 2 HFs accounted for 57.7% of all HF encounters, averaging 421 encounters monthly, whereas fewer but larger level 3 and 4 HFs accounted for 697 and 569 monthly encounters, respectively. Suspected malaria cases comprised 81.0%, 60.9%, and 30.2% of encounters at Levels 2, 3, and 4 HFs, respectively. HF encounters peaked annually in June–July during the malaria transmission season, while CHV encounters showed minimal month-to-month variation (Fig. 3).

CHVs had greater utilization in 83.2% (415/499) of villages with inadequate HF coverage, averaging 313.6 vs. 224.8 encounters per 1,000 population in villages adequately vs. inadequately covered by HFs, underscoring their role in healthcare access.

### Malaria consultations

Malaria testing volumes increased steadily coinciding with the project’s phased rollout and enhanced supervision, peaking in 2023, with averages of 71.5, 97.7, and 87.8 malaria tests (RDT and blood smears) per week in Levels 2, 3, and 4 HFs, respectively. In the same year, each CHV averaged 3.1 RDT tests per week (95% CI 3.0–3.3), accounting for 22.8% (SD = 11.2) of all CHV services and 12.4% (95% CI 11.0–12.9) of total malaria tests. HF test volumes showed greater week-to-week variability than did CHVs (Fig. 3).

**Fig. 4:**
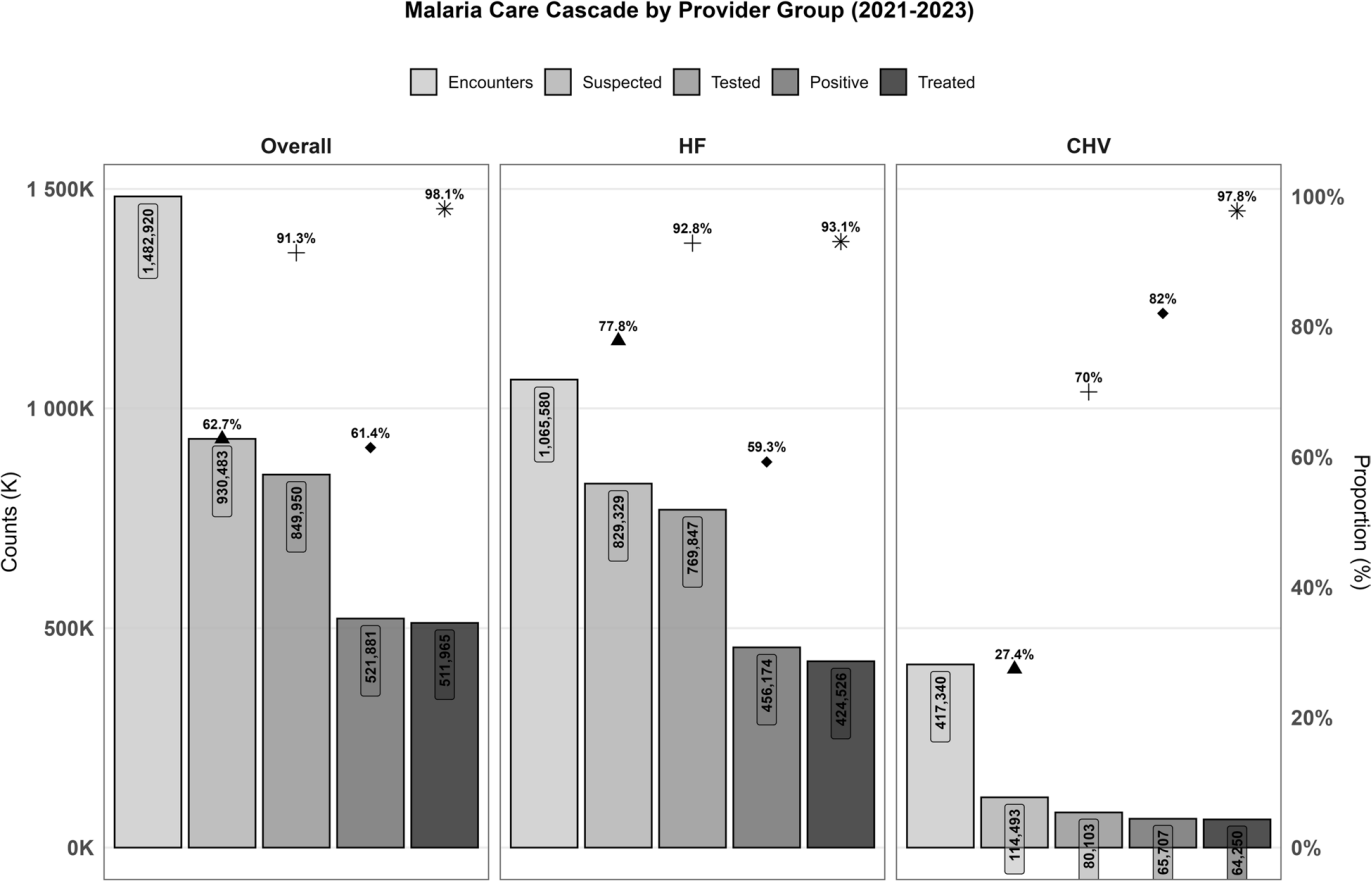
Malaria care cascade by provider group in Alego Usonga and Rarieda Sub County, Siaya (2021–2023). The cascade shows the number of malaria encounters progressing through suspected, tested, confirmed positive, and treated for all providers combined (Overall), health facilities (HF), and community health volunteers (CHV). Bar heights indicate absolute counts, while the percentage represent transition between stages of care. Health facility data include both rapid diagnostic tests (RDT) and blood smear (BS) testing

Of the 1,482,920 healthcare encounters, 28.1% were managed by CHVs. Suspected malaria accounted for 63.6% of all encounters, with HFs managing 87.8% of these cases. Overall, 91.3% of the suspected malaria cases were tested, primarily with RDTs (74.5%, n = 633,373), whereas blood smears (BS), which are exclusive to HFs, comprised 28.1% of the HF tests (Table 2, Fig. 4).

**Table 2:** Summary of encounters and demographic characteristics of individuals tested by community health volunteers and health facilities, Siaya^#^, Kenya (2021–2023)

Category	CHV (N = 417,340)	HF (N = 1,065,580)	Overall (N = 1,482,920)	p-value^g^
I. Suspected and tested cases				
Suspected Malaria	114,493 (27.4%)	829,329 (77.8%)	930,483 (62.7%)	< 0.001
Febrile^a^	77,923 (77.0%)	440,581 (53.1%)	518,504 (55.7%)	< 0.001
Tested for malaria	80,103 (70%)	769,847 (92.8%)	849,950 (91.3%)	< 0.001
RDT^a^	80,103 (100%)	553,270 (71.9%)	633,373 (74.5%)	< 0.001
BS	–	216,577 (28.1%)	216,577 (28.1%)	-
Febrile tested	57,832 (74.2%)	389,766 (88.5%)	447,598 (86.3%)	< 0.001
II. Test positivity				
Any positive test^b^	65,707 (82%)	456,174 (59.2%)	521,881 (61.4%)	< 0.001
RDT positive^c^	65,707 (82%)	354,767 (64.1%)	420,474 (66.4%)	< 0.001
BS positive^d^	–	101,407 (46.8%)	101,407 (46.8%)	-
Treated with ACT^e^	64,250 (97.4%)	424,526 (95.2%)	511,965 (95.5%)	< 0.001
Confirmed cases (RDT + BS)	63,636 (99.0%)	419,740 (98.9%)	483,376 (98.9%)	< 0.001
BS positive cases	-	94,647 (93.3%)	94,647 (93.3%)	-
Treated without testing^f^	1,729 (5.0%)	21,460 (7.3%)	23,189 (4.5%)	< 0.001
Febrile	769 (44.5%)	12, 793 (59.6%)	13,562 (58.5%)	< 0.001
III. Demographics (RDT Tested Only)				
Age, years (SD)*	16.4 (17.4)^*^	18.1 (17.6)	17.9 (17.6)	< 0.001
Age group*				
0–5 years	24,544 (30.6%)	136,650 (24.7%)	161,194 (25.5%)	< 0.001
5–14 years	28,012 (35.0%)	172,928 (31.3%)	200,940 (31.7%)	
15 + Years	27,547 (34.4%)	243,692 (44.0%)	271,239 (42.8%)	
Sex: Female*	40,841 (56.0%)	314,008 (58.7%)	354,849 (58.4%)	< 0.001
Year*				
2021	12,812 (16.0%)	125,137 (22.6%)	137,949 (21.8%)	< 0.001
2022	26,326 (32.9%)	181,167 (32.7%)	207,493 (32.8%)	
2023	40,965 (51.1%)	246,966 (44.6%)	287,931 (45.5%)	

Suspected malaria is defined as encounters with any of the following: (i) fever or a history of fever within the past 7 days; or (ii) tested for malaria; or (iii) treated with artemisinin-based combination therapy (ACT).

*Data for Demographics pertain only to individuals tested for malaria using RDT

^a^Denominator is suspected malaria

^b^Denominator is the total number of tested (RDT + BS)

^c^Denominator is the total number of RDTs tested

^d^Denominator is the total number of BS tested

^e^Denominator is the total number of suspected malaria cases tested

^f^Denominator is the total number of suspected malaria cases not tested

^g^p-values calculated using Chi-square test

^#^ In two sub counties-Alego Usonga and Rarieda

Compared with CHVs, HFs accounted for a greater proportion of suspected cases and a greater proportion of febrile cases [92.8% vs. 79.2% and 88.5% vs. 74.2%, respectively]; testing rates were similar among nonfebrile cases (60.9% vs. 60.8%). The mean age of the tested patients was 17.9 years (SD = 17.6), with 25.5% under 5 years, 31.7% aged 5–14 years, and 42.8% aged ≥ 15 years (Table 2). A greater proportion of suspected cases visiting CHVs reported fever than HFs (77.0% vs 53.1%). The overall TPR was 61.3%, RDT TPR was 66.5% (HF + CHV), and BS TPR was 46.2% (HF only). RDT positivity was higher among CHV-tested patients (82.0%) than among HFs (64.3%, p < 0.001), which was consistent among both febrile (83.6% vs. 72.4%, p < 0.001) and afebrile patients (77.9% vs. 53.2%, p < 0.001) (Table 3). TPR was highest in children aged 5–14 years (77.3%), followed by those under 5 years (69.5%). When stratified by provider, TPR was highest among < 5yrs tested by CHVs (85.5%) and among 5-14yrs tested at HFs (76.0%). CHVs administered ACTS to 97.4% (n = 64,250) of suspected cases tested compared to 95.2% (n = 424,526) by HFs (p < 0.001). Of these, nearly all confirmed malaria cases (98.9%) received ACTs (99.0% for CHVs; 98.9% for HFs). Among BS-positive cases assessed at HFs, 93.3% (n = 94,647) received ACTs. CHVs accounted for 5.0% of the suspected cases not tested (n = 1,729: 3.8% febrile, 6.7% afebrile), whereas HFs accounted for 7.3% (n = 21,460: 25.2% febrile, 3.5% afebrile; p < 0.001) (Table 2).

**Table 3:** RDT positivity rates conducted by community health volunteers and at health facilities in Siaya^#^, 2021–2023

Characteristic	Category	Community health volunteers			Health facility		
		Positive (%)	Negative (%)	P value	Positive (%)	Negative (%)	P value
Sex	Female	33,007 (80.8%)	7,834 (19.2%)	< 0.001	192,417 (61.3%)	121,591 (38.7%)	< 0.001
	Male	26,871 (83.6%)	5,267 (16.4%)		150,048 (68.0%)	70,623 (32.0%)	
Age group (years)	0-5yrs	20,993 (85.5%)	3,551 (14.5%)	< 0.001	90,964 (66.6%)	45,686 (33.4%)	< 0.001
	5-14yrs	23,738 (84.7%)	4,274 (15.3%)		131,496 (76.0%)	41,432 (24.0%)	
	15 + yrs	20,976 (76.1%)	6,571 (23.9%)		132,307 (54.3%)	111,385 (45.7%)	
Year	2021	10,648 (83.1%)	2,164 (16.9%)	< 0.001	78,509 (62.7%)	46,628 (37.3%)	< 0.001
	2022	21,796 (82.8%)	4,530 (17.2%)		121,667 (67.2%)	59,500 (32.8%)	
	2023	33,263 (81.2%)	7,702 (18.8%)		154,591 (62.6%)	92,375 (37.4%)	
Subcounty	Alego Usonga	25,809 (86.0%)	4,205 (14.0%)	< 0.001	226,716 (68.0%)	106,650 (32.0%)	< 0.001
	Rarieda	39,898 (79.7%)	10,191 (20.3%)		128,051 (58.2%)	91,853 (41.8%)	
Fever	No	17,347 (77.9%)	4,924 (22.1%)	< 0.001	126,528 (53.2%)	111,527 (46.8%)	< 0.001
	Yes	48,360 (83.6%)	9,472 (16.4%)		228,239 (72.4%)	86,976 (27.6%)	

^#^in two sub counties–Alego Usonga and Rarieda, with valid RDT results

P value for Chi-square test

Percentage (%) in each cell is computed using the total number of nonmissing records as the denominator. In some cases, the participants had missing data for the covariates

### Factors affecting malaria RDT positivity

Modified Poisson regression identified individual-level factors associated with RDT positivity, including higher risk among children aged 5–14 years, males, and individuals with a history of fever. HF-tested encounters were less likely to be RDT positive than CHV-tested encounters (aRR = 0.81, 95% CI 0.79–0.82), and individuals aged 15 + years also had a reduced likelihood of positivity (Table 4). Positivity rates varied temporally and geographically, with elevated risk from 2021–2022 and across subcounties. Village-level predictors, such as CHV and HF coverage, showed no significant associations (Table 4).

**Table 4:** Multivariate analysis of risk factors for RDT positivity in Malaria Surveillance, Siaya County, Western Kenya (2021–2023)

Factors	aRR* (95% CI)		P-value
Age group (years)	0-5yrs	Ref	< 0.001*
	5-14yrs	1.14 (1.13, 1.15)	
	15 + yrs	0.82 (0.81, 0.84)	
Provider type	CHV	Ref	< 0.001*
	Health Facility	0.81 (0.79, 0.82)	
Sex	Female	Ref	< 0.001*
	Male	1.06 (1.05, 1.06)	
Fever	No	Ref	< 0.001*
	Yes	1.29 (1.26, 1.32)	
Sub County	Alego Usonga	Ref	< 0.001*
	Rarieda	0.88 (0.86, 0.9)	
Year	2023	Ref	
	2021	1.03 (1.01, 1.06)	0.007
	2022	1.07 (1.05, 1.08)	< 0.001*
CHV coverage^†^	Adequate	Ref	0.246
	Inadequate	0.98 (0.96, 1.01)	
HF coverage^†^	Adequate	Ref	0.133
	Inadequate	0.98 (0.96, 1.01)	

^*1*^ aRR – adjusted Risk Ratio, with 95% confidence intervals

P-value from z-test using clustered standard errors

Ref – reference category

^†^Cluster-level predictors defined at the village level. Standard errors were adjusted for clustering; all other predictors are measured at the individual level

Distance and accessibility scores were excluded from the final model

### Incidence rates

In 2023, malaria incidence was higher when CHV data were included: 549 vs. 467 cases per 1000 population for CHV + HF vs. HF-only reporting (mean difference of 81.5 cases per 1000, p < 0.001 by Shapiro‒Wilk and Wilcoxon signed-rank tests), suggesting potential underestimation in HF-only data. The TPRs were also greater in the combined CHV + HF group. A mixed-effects model estimated an adjusted difference of 81 cases per 1,000 (β = –81.1, p < 0.001), reinforcing improved case detection with CHV reporting. Adequate CHV coverage was associated with increased village-level incidence: + 108 cases per 1,000 in the CHV + HF model (p = 0.002) vs. + 97 in the HF-only model (p = 0.004). Adequate HF coverage was also significantly associated with a higher incidence in both models (+ 188 and + 205 cases per 1,000, respectively; p < 0.001).

## Discussion

This study highlights the vital role of CHVs in malaria case detection and management, especially in underserved areas. Although nearly all villages met the WHO-HF coverage standard (≤ 5 km from a HF), only 30% were classified as adequately covered using modeled HF accessibility scores that integrate distance, utilization and catchment population. This suggests that geographic proximity alone does not ensure effective coverage, as quality, costs, and trust may influence care-seeking. In contrast, 82% of the villages met the CHV coverage standards. CHVs helped bridge coverage gaps, ameliorating some of the effects of inadequate coverage and low utilization of HFs. With an average of one CHV per375 residents—well above the MOH standard (1:500)—their deployment mirrors successful models in Southeast Asia, where village malaria workers strategically extended services to hard-to-reach areas and reduced malaria incidence through diagnosis, treatment, and surveillance [18, 44]. Combined with their strong community presence, CHVs foster trust, encouraging care seeking and utilization of health services. Integrating CHV deployment strategies with accessibility models such as 2SFCA may enhance service coverage and expand reach, particularly in low HF utilization regions.

### Potential for reducing the burden in health facilities through task shifting

While CHVs managed 12.4% of all malaria testing, they accounted for 28.1% of all healthcare encounters in the study area, underscoring their contribution beyond malaria-specific care. This level of engagement positions CHVs as a core tier of primary care delivery. By managing over one-quarter of encounters, they operationalize task shifting of uncomplicated malaria case management and expand community-level service delivery. The surveillance initiative strengthened reporting and supervision, enhancing visibility of CHV contributions. A greater proportion of patients seeking care from CHVs reported fever, and TPRs were high across all patient groups, suggesting a tendency for patients to consult CHVs when malaria is suspected. CHV treatment practices closely align with national malaria guidelines, with nearly all confirmed malaria cases receiving ACTs. This underscores their critical role in early case detection [45] and highlights how, despite the relatively smaller caseload, CHVs are essential in alleviating the malaria burden at HFs. Equipping them to test more cases at the community level could help shift the substantial burden of suspected malaria cases away from HFs, allowing HFs to focus on more severe cases and other diseases and improving healthcare efficiency. This task-shifting approach—effective in South Africa and Zambia [46, 47]—requires strong CHV capacity, reliable access to RDTs and ACTs, and enhanced supervision and robust monitoring systems to maintain care quality and ensure high-quality data [6, 11, 13, 30, 31, 47]. Future research should enablers of effective CHV integration and system-level adjustments needed to support broader roles in case management.

While HFs tested a greater proportion of suspected cases, CHVs were more likely to provide ACTs to RDT-positive cases. Although stockouts of RDTs may have contributed to the lack of testing by CHVs, only a small proportion of untested patients received treatment for malaria (3.8% of febrile and 6.7% of afebrile patients). In instances where CHVs lack both RDTs and ACTs or when patients present with danger signs or a history of recent ACT use, CHVs are instructed to refer patients to HFs. However, the CHV activity register does not capture additional symptoms (besides fever) that may warrant testing, danger signs, or referral actions. This omission makes it challenging to assess the degree to which CHVs adhere to national guidelines for untested patients. Incorporating additional fields to document other symptoms, danger signs and referrals on the activity register would enhance the evaluation of adherence to guidelines.

### CHV data for public health planning

CHV-testing was associated with higher TPRs and inclusion of CHV-reported cases increased malaria incidence estimates from 467 to 549 per 1,000 compared with HF-only, indicating enhanced case detection. Beyond enhancing surveillance accuracy, identifying and treating these cases may reduce the parasite reservoir and potentially lower transmission, although this was not directly measured. Such enhanced detection may also support community-based surveillance of febrile illnesses, early warning systems, and iCCM. However, underestimation of the true malaria burden persists because patients are treated in nonformal settings such as private pharmacies. Beyond incidence, CHVs’ granular household-level census data—such as demographics and population sizes of households, villages, and CUs—could better inform public health workforce planning [48]. As shown in Uganda and Liberia, integrating CHV data into national surveillance platforms such as the district health information software (DHIS2) improves data-driven decision making, strengthening surveillance and intervention targeting [6, 11, 30, 31]. By leveraging CHV data, malaria endemic countries could allocate resources more effectively by targeting areas of greatest need.

### Limitations

This study faced methodological and data-related constraints. First, coverage assessments based on population metrics and travel distances may not fully capture the complexities of CHV and HF coverage comparisons. While it accounts for distances, it assumes uniform geographic barriers–such as rivers, swamps and mountains–and consistent service delivery across provider types, which may oversimplify care-seeking patterns [49, 50]. The 2SFCA method and Gaussian decay function provide a static view of accessibility and may overlook temporal variation, real-world complexities and behavioral nuances of care seeking [51, 52].

Data exclusions further limited the scope: HF attendees missing village-of-residence data were excluded because of concerns about quality or residing outside the study area, although this represented only a small proportion of all encounters. HF operational capacity–including staffing and commodity availability–was not assessed, potentially misrepresenting service delivery [53]. Comorbidities in HF data were not analyzed, possibly inflating suspected cases of malaria [54].

## Conclusion

This study offers robust evidence of the critical role of CHVs in malaria case management in Siaya County, western Kenya. CHVs managed 28.1% of all encounters and 12.4% of malaria cases across all patient groups, demonstrating their value in delivering CCM. Furthermore, incorporating CHV-reported cases improved malaria incidence estimates and strengthened case detection. To maximize their impact, strategic scale-up with sufficient RDT and ACT supplies, effective supervision, and better remuneration, could improve CHV retention and service delivery, thereby reinvigorating stalled control efforts. Future initiatives should prioritize strategic deployment of CHVs and integration of private sector data to strengthen community-based care.

## Supplementary Information

Supplementary Material 1.

Supplementary Material 2.

Supplementary Material 3.

Supplementary Material 4.

Supplementary Material 5.

Supplementary Material 6.

## Data Availability

The data supporting the findings of this study are available from the Kenya Medical Research Institute–Center for Global Health Research (KEMRI‑CGHR). Access is subject to Kenya data protection regulations. Data may be obtained upon reasonable request and with approval from the KEMRI Scientific and Ethics Review Unit (SERU) and the project investigators.
