# Supplementary figures and images for: Community health volunteers improve access to malaria case management in Siaya County, Kenya
Source: Malar J. 2026 Mar 31;25:202. doi: 10.1186/s12936-026-05883-3 (PMC13162475; doi:10.1186/s12936-026-05883-3)

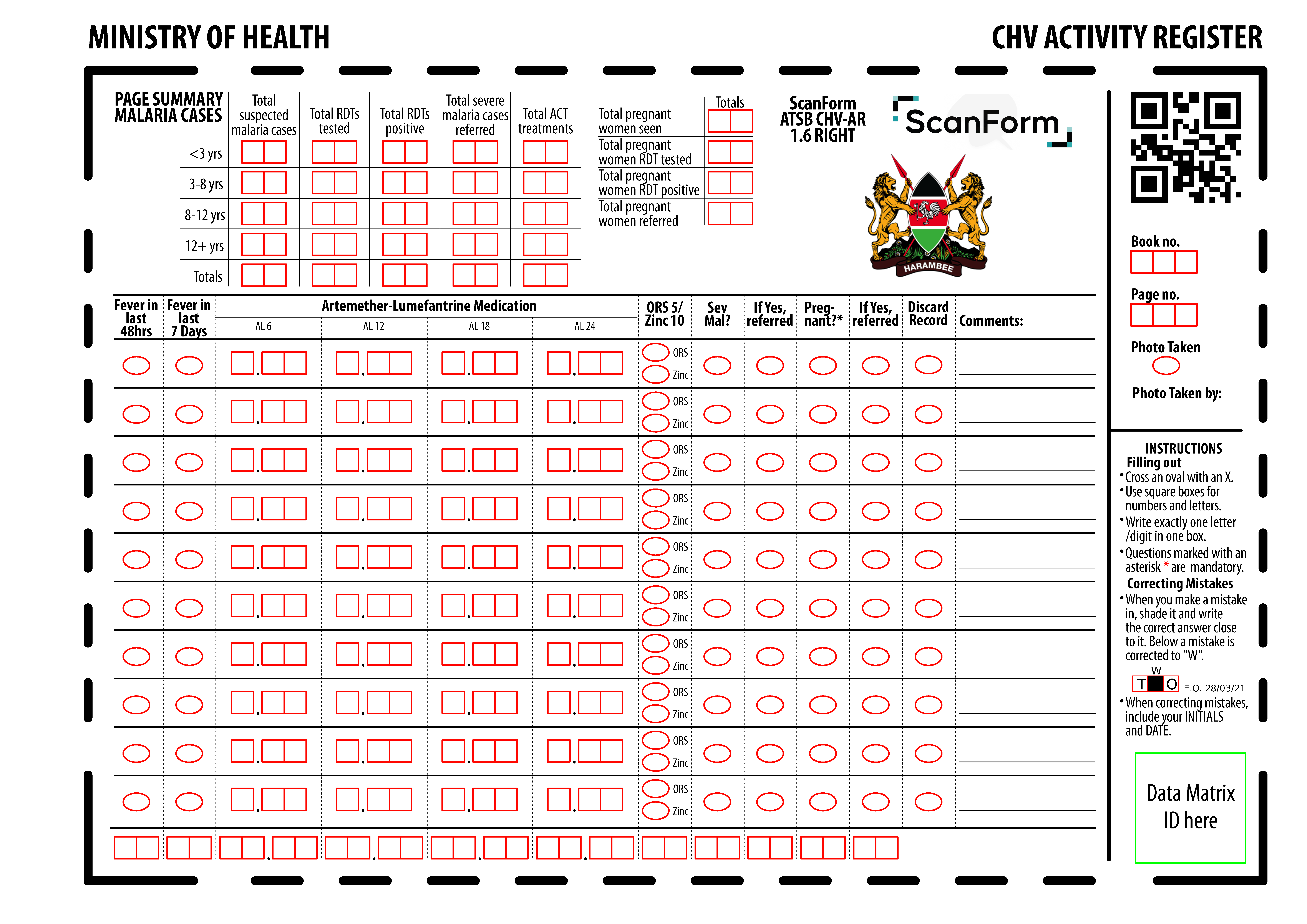

Supplement: Supplementary file 2 — Supplementary Material 2. [file 12936_2026_5883_MOESM2_ESM.tif]

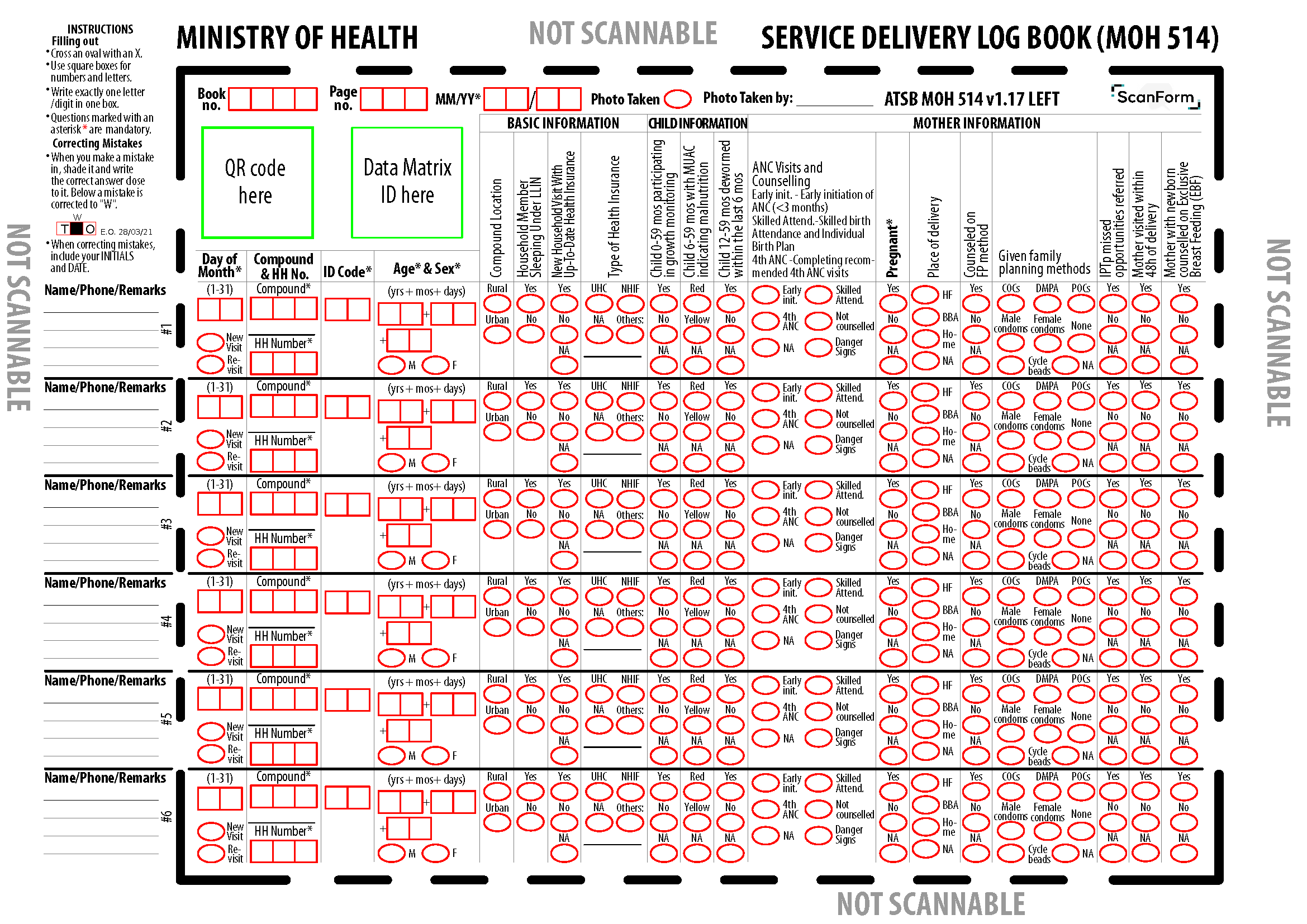

Supplement: Supplementary file 3 — Supplementary Material 3. [file 12936_2026_5883_MOESM3_ESM.tif]

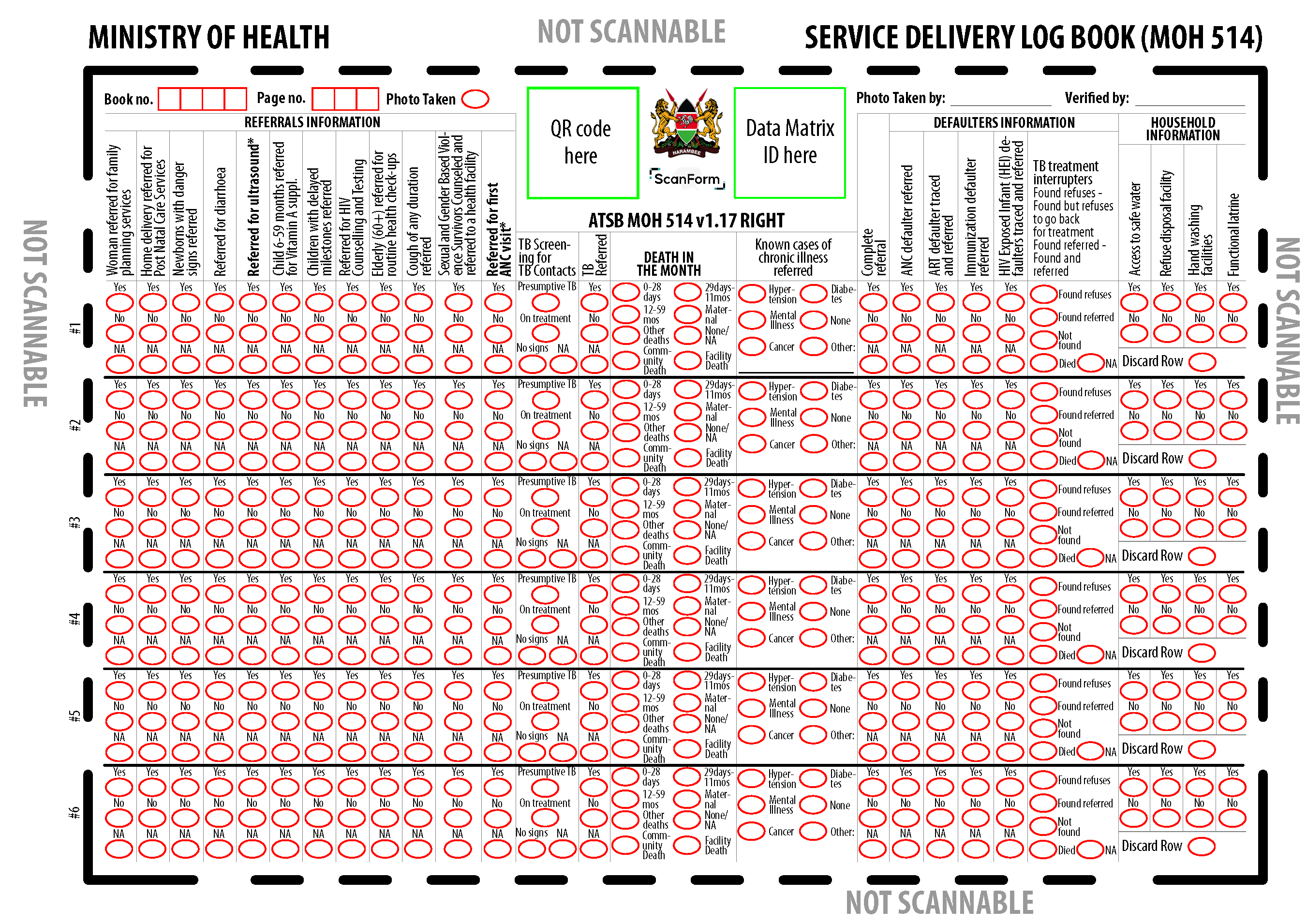

Supplement: Supplementary file 4 — Supplementary Material 4. [file 12936_2026_5883_MOESM4_ESM.tif]

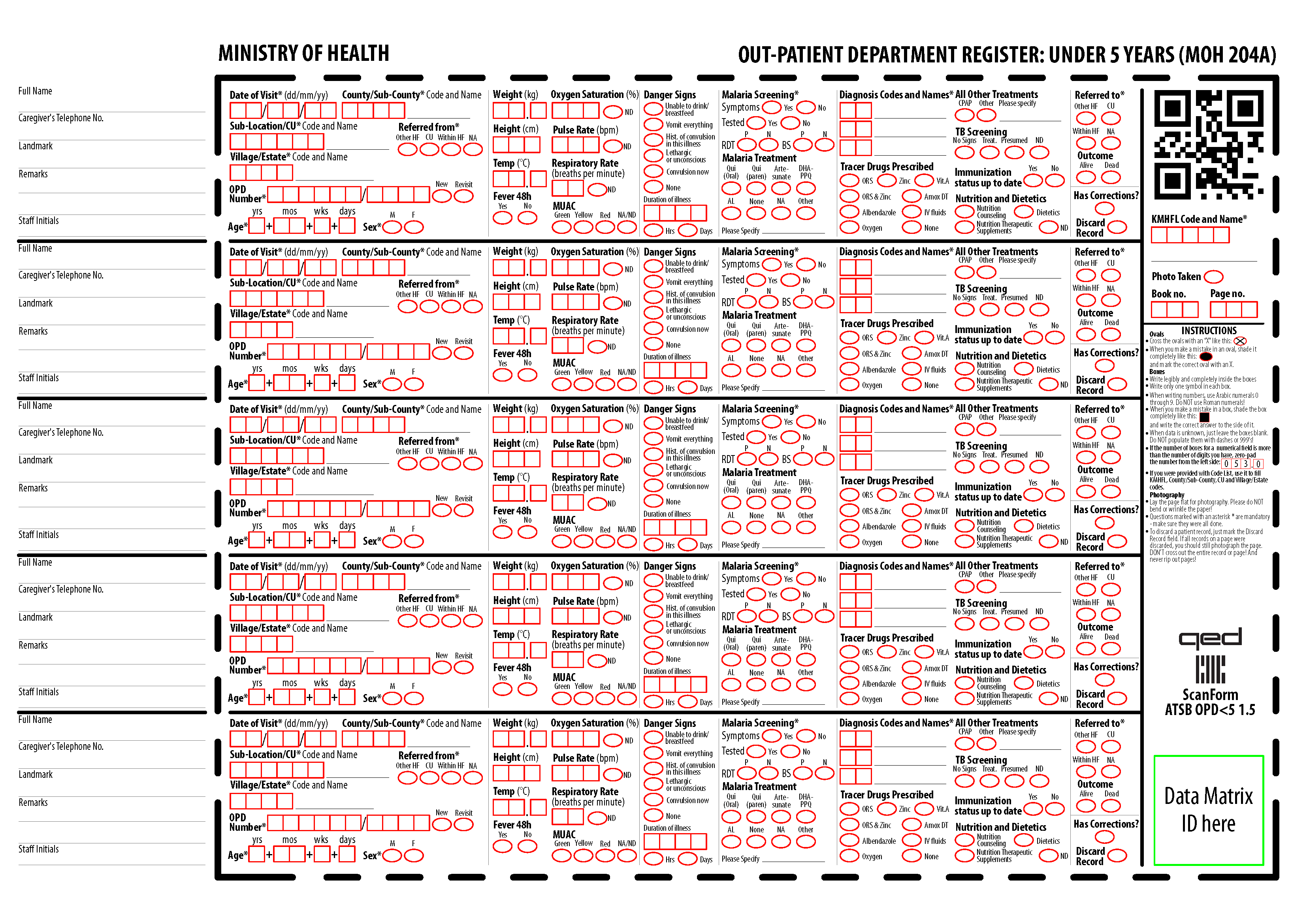

Supplement: Supplementary file 5 — Supplementary Material 5. [file 12936_2026_5883_MOESM5_ESM.tif]

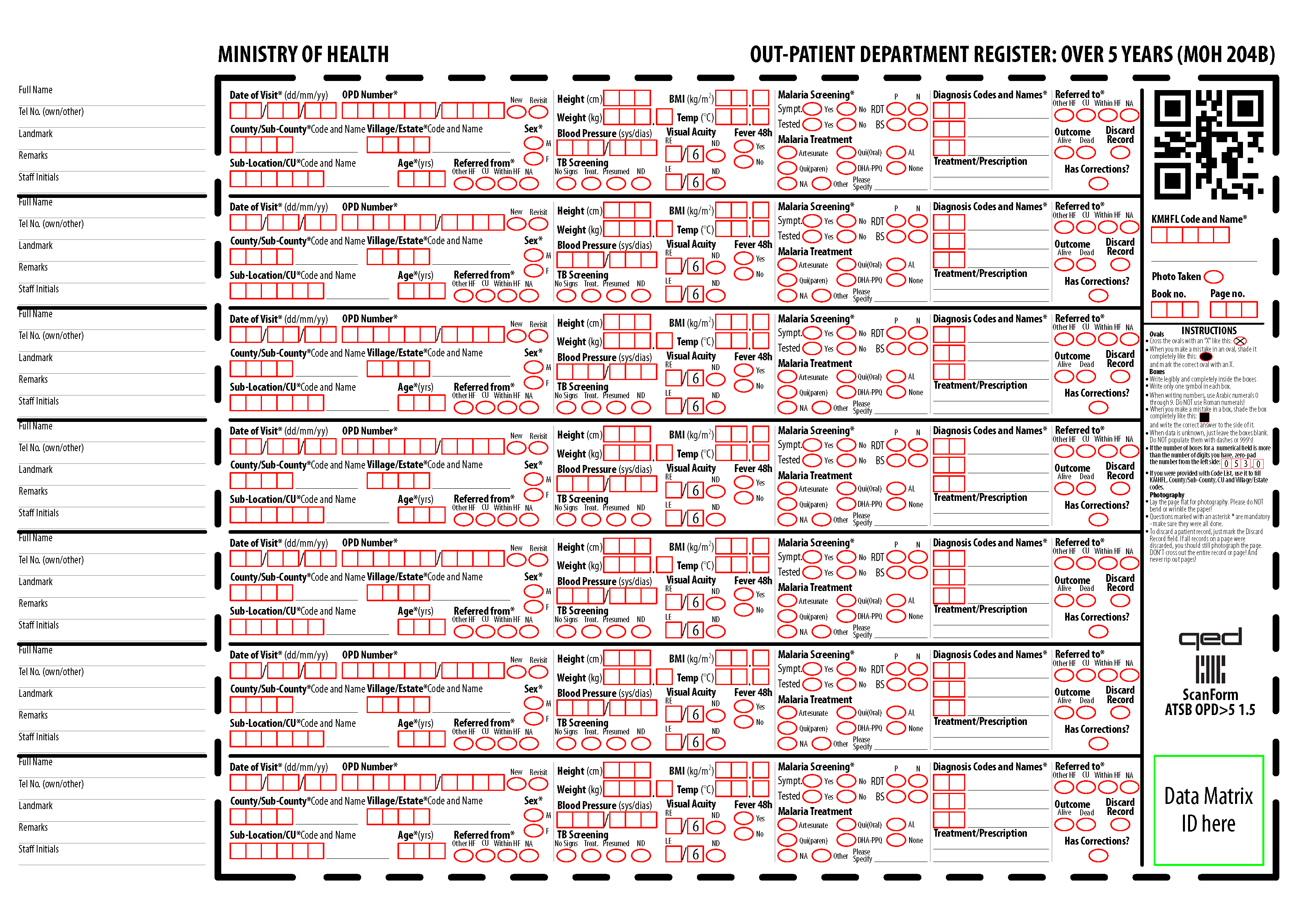

Supplement: Supplementary file 6 — Supplementary Material 6. [file 12936_2026_5883_MOESM6_ESM.tif]
